# Axial Tensile Ultimate Strength of an Unbonded Flexible Riser Based on a Numerical Method

**DOI:** 10.3390/ma17102286

**Published:** 2024-05-11

**Authors:** Dongya Li, Wanchao Jiang, Qingqing Xing, Qingsheng Liu

**Affiliations:** 1 Applied Technology College of Soochow University, Suzhou 215000, China; jiangwanchao97@gmail.com (W.J.); qqxing@suda.edu.cn (Q.X.); 2 School of Mechanical and Electric Engineering, Soochow University, Suzhou 215131, China; qsliu@suda.edu.cn

**Keywords:** unbonded flexible riser, tensile armor layer, axial tensile stiffness, axial tensile strength, frictional coefficient

## Abstract

Unbonded flexible risers consist of several helical and cylindrical layers, which can undergo large bending deformation and can be installed to different configurations to adapt to harsh marine environments, and is a key equipment in transporting oil and gas resources from Ultra Deep Waters (UDWs) to offshore platforms. The helical interlayer of an unbonded flexible riser makes the structural behavior difficult to predict. In this paper, the axial tensile behavior and the axial tensile ultimate strength of an unbonded flexible riser are studied based on a typical 2.5-inch eight-layer unbonded flexible riser model, and verified through a theoretical method considering the contact between adjacent layers. First, the balance equation of separate layers is deduced by a functional principle, and then the overall theoretical model of an unbonded flexible riser is established considering the geometric relationship between adjacent layers. Then, the numerical model considering the detailed geometric properties of an unbonded flexible riser is established to simulate the axial tensile behavior. Finally, after being verified through the experimental results, the axial tensile stiffness and axial tensile strength of an unboned flexible riser considering the elasticity of the tensile armor layer are studied using the proposed two methods. Additionally, the effect of frictional coefficients is conducted. The numerical and theoretical results show good agreement with the test results, and the friction between adjacent layers would increase the axial tensile stiffness of an unbonded flexible riser.

## 1. Introduction

**Figure 1:**
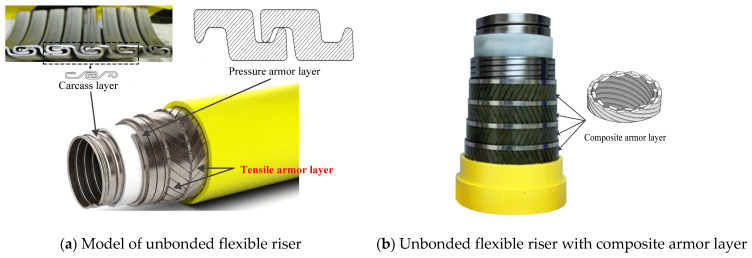
Sketch of unbonded flexible riser.

Unbonded flexible risers consist of multilayers, and the structure forms can be flexibly arranged according to the needs of production and operation, which have wide applications in the ocean engineering industry field. The special construction of the riser structure allows for low bending stiffness while maintaining sufficient axial tensile stiffness, and has become an important equipment for the extraction of oil and gas resources from Ultra Deep Waters (UDWs). A typically sketch of an unbonded flexible riser model is presented in Figure 1. With the development of offshore industries towards Ultra Deep Waters, higher requirements are put forward for the offshore engineering equipment. Some other functional special layers are also common in an unbonded flexible riser model, like anti-birdcage tape [1], an anti-H_2_S layer [2], an insulation layer and even cables, etc. In order to reduce the mass of the suspended section of an unbonded flexible riser, and to strengthen the cross-sectional properties, composite armor layers are proposed [3,4,5,6]. During the long production cycle of an unbonded flexible riser, riser failure occurs from time to time, which may cause major environmental accidents such as oil spills at sea, and bring immeasurable economic and environmental losses. Thus, a numerical method with detailed the geometric properties of an unbonded flexible riser is proposed in this paper to study the axial tensile behavior and the corresponding axial tensile strength of an unbonded flexible riser.

The separate interlayer of an unbonded flexible riser is not bonded and can slip somehow. Thus, the complex geometric properties and the material properties of the riser lead to the complex cross-sectional properties of an unbonded flexible riser. The internal helical layers (mainly the tensile armor layer) would undergo relative slippage subjected to different loading modes, which leads to asynchrony between the excitation and the response due to friction, and the hysteresis phenomenon occurs.

Theoretical and numerical methods are commonly used to analyze the structural response characteristics of an unbonded flexible riser, since a test specimen of an unbonded flexible riser is typically expensive and some specialized experimental facilities are also sometimes required for the simulation of external loads.

The theoretical approach is usually to establish the force equilibrium equations of each separate layer, and then establish the overall analytical model through the geometric relationship between adjacent layers. The response of helical layers within an unbonded flexible riser is the key to solving the cross-sectional mechanical properties of an unbonded flexible riser. Féret and Bournazel [7] present an analytical method to quickly assess the stress of the helical tendon, while ignoring the effects of internal and external pressures and interlayer gaps in an unbonded flexible riser model, and it is concluded that the non-metallic cylindrical shell layer only transmits the interlayer pressures and ignores the role of its axial stiffness. Berge et al. [8] proposed a fast method for calculating the overall response of an unbonded flexible riser, and gave expressions for the stress–load relationship under separate actions of axial force, internal pressure and torque, respectively, but the theoretical method is only applicable to separate load actions, and the response characteristics of each layer cannot be decoupled. Kebadze and Kraincani [9,10] made a great contribution to the theoretical modeling of unbonded flexible risers under axisymmetric loading, and they summarized the theoretical models of their predecessors by dividing all the layers of unbonded flexible risers into cylindrical shell layers and helical layers; assuming that each layer has the same axial elongation and torsion angle, and considering the axial elongation and torsion angle of the overall unbonded flexible riser, as well as the thickness and radial strain of each layer, combined with the geometric relationship between the layers, the overall stiffness matrix is deduced through the functional principle; this can be used to arbitrarily solve the unknowns of each layer according to the force condition, determine the contact condition of neighboring layers and calculate the effect of the interlayer contact pressure. Dong et al. [11] fully considered the effect of local bending and torsional deformation in the helical tendon, and improved Kebadze’s model. Liu et al. [6] extended the theoretical model of the steel tensile armor layer to the model of the composite tensile armor layer, which can be used to calculate the structural response of unbonded flexible risers containing both a steel tensile armor layer and a composite tensile armor layer under axisymmetric loading. Numerical methods have evolved from equivalent simplified models to models that account for detailed geometric properties. Additionally, Sathikh [12] derived the linear elastic models of a helical wire under axisymmetric loads. Karathanasopoulos [13] studied the effect of thermal loads on the helical constructions. Current research mainly focuses on how to accurately describe the slip characteristics of the helical tendon, including the tensile armor layer bending hysteresis model established by considering different friction models and based on the theory of bending beams [14,15]. An analytical analysis model considers the deformation characteristics of the tensile armor layer under the action of riser torque and bending around the axis [9]; the bending hysteresis model was established by considering the effect of shear deformation of the cylindrical shell layer structure on the riser [16].

Most of the earlier numerical models of unbonded flexible risers provide some simplification of the complex internal structure or create new elements through the secondary development method [17,18]. Sousa et al. [19,20] carried out many studies on the mechanical properties of unbonded flexible risers based on numerical methods; he simplified the carcass layer and pressure armor layer, which have complex cross-sectional properties, into orthogonal anisotropic shell units, and simulated the tensile armor layer by isotropic three-dimensional Euler beam unit. And the above numerical model can be used to analyze the cross-sectional mechanical properties of an unbonded flexible riser under axisymmetric loads such as axial force and external pressure. Bahtui et al. [21,22] established the actual shape of the tensile armor layer based on ABAQUS software (https://www.3ds.com/products/simulia/abaqus, accessed on 7 May 2024) by using an eight-node linear reduced integrator unit for simulations and took into account the contact effect between adjacent layers. With the improvement in computer computational performance, more and more scholars began to consider the establishment of numerical models containing the detailed geometric characteristics of unbonded flexible risers. Ren et al. [23,24] established a numerical model of an eight-layer unbonded flexible riser based on ABAQUS software considering the establishment of an S-type carcass layer and a Z-type pressure armor layer, which contain all the geometric characteristics, and all the layer structures were simulated with eight-node linear reduced integral body units. The proposed numerical can effectively simulate the inter-layer and intra-layer contact and friction, and therefore can be used to calculate the cross-sectional mechanical properties of an unbonded flexible riser under axisymmetric and bending loads. Zhang et al. [25] also developed a numerical model containing detailed geometries, and all layers of the structure were also simulated using body cells to analyze the structural response under the combined effects of external pressure and bending moments. Yoo et al. [26,27] established a carcass layer with detailed geometrical characteristics through ANSYS software (https://www.ansys.com/, accessed on 7 May 2024), and simplified it into an equivalent cylindrical shell layer by analyzing the mechanical failure characteristics of the carcass layer under the action of axial force, and established an equivalent simplified eight-layer numerical model and a five-layer numerical model; at the same time, they considered the effect of the shell unit or the body unit in simulating the cylindrical shell layer, and investigated the axial load-bearing capacity of the unbonded flexible riser. Liu et al. [6,28] established a local column coordinate system to define the material properties of the composite armor layer within ABAQUS software, and studied the behavior of an unbonded flexible riser under coupling loads. The development of numerical models of unbonded flexible risers containing detailed geometric properties to simulate the nonlinear hysteresis characteristics of the structure is the current direction of development [29,30,31,32].

The aim of this paper is to analyze the axial tensile behavior of an unbonded flexible riser and the fracture failure of the tensile armor layer based on a numerical method considering the detailed geometric properties of an unbonded flexible riser; this is verified through a theoretical method. Based on a typical 2.5-inches eight-layer unbonded flexible riser model, the numerical model containing all the geometric properties of an unbonded flexible riser is modeled within ABAQUS software and the ratio of kinetic energy to internal energy is very small to ensure calculation accuracy. And, then, taking the deformation characteristics of the helical layer and cylindrical layer under axisymmetric loading into account, the balance equation of each layer considering the axial, radial and circumferential strains and the torsional angle is established according to the functional principle, and the corresponding geometric relationship between adjacent layers is also included to build up the overall theoretical model of an unbonded flexible riser. Afterwards, the tensile behavior of an unbonded flexible riser and the fracture of the tensile armor layer under axial tension is investigated by the proposed methods and the effect of the frictional coefficient is also considered in this paper.

## 2. Theoretical Formulations

**Figure 2:**
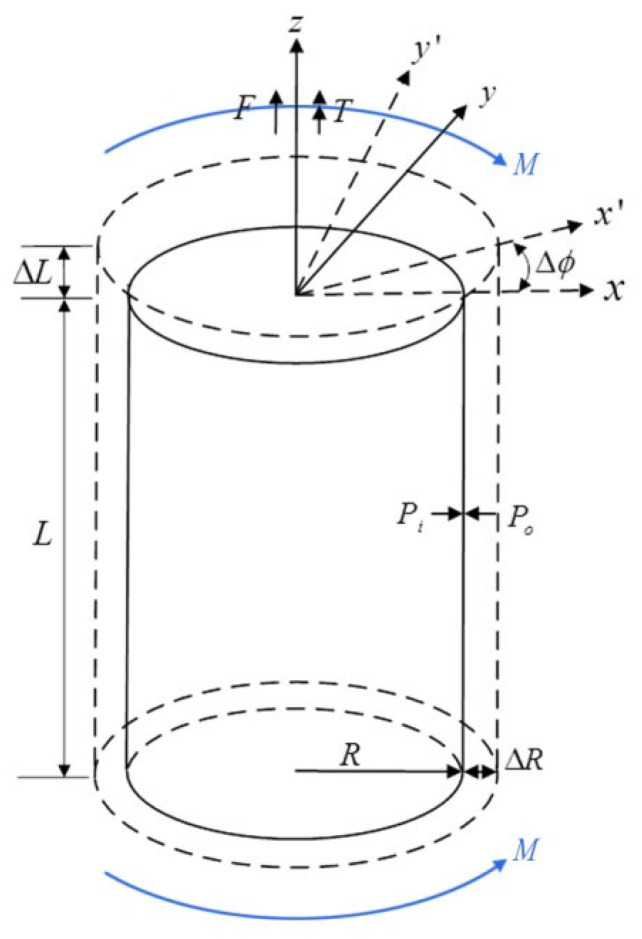
Forced and deformation sketch of unbonded flexible riser model under complex external loads [28].

We assume that the deformation of an unbonded flexible riser under external loads can be simplified as axisymmetric loads (tension, torsion, internal and external pressure) and bending moment; please see Figure 2. Some assumptions have to be introduced before the theoretical model of an unbonded flexible riser is developed:

1. The external load on an unbonded flexible riser can be decoupled into axisymmetric loads and bending moment;
2. When studying the cross-sectional mechanical properties of an unbonded flexible riser, all consisted layers are assumed to be in the small deformation stage and we neglect the material nonlinearity;
3. The riser has a sufficient length (*L*/*D* ≅ 10, where *L* is the initial length of the riser and *D* is the outer diameter of the riser), and the end-effect is neglected;
4. The effect of the bending stiffener is neglected and the initial imperfection of the riser is also neglected; we assume that each layer of the riser has the same axial elongation and torsion angle along the central axis;
5. For helical layers, the frictional energy by the slippage of the helical tendon is neglected;
6. The anti-friction layer is simplified as a cylindrical layer;
7. The thickness deformation of the carcass layer, as well as the pressure armor layer, is neglected;
8. When studying the fracture of the tensile armor layer, we consider that the tensile armor layer loses its bearing capacity when it reaches the yield stress.

### 2.1. Theoretical Model of Cylindrical Layer under Axisymmetric Loads

It can be seen in Figure 2 that the axisymmetric loads include the axial force *F*, torsion *T* and the internal and external pressure *P_i_* and *P*_o_, neglecting the effect of the bending moment and assuming that the energy exerted by the axisymmetric loads can be given by the following equation [9,24,33]:

(1) $$W=F\Delta L+T\Delta \varphi +P_{i}\Delta V_{i}-P_{o}\Delta V_{o}$$

where ΔL is the axial deformation, Δφ is the rotational angle about the axial direction of unbonded flexible riser and $\Delta V_{i}$ and $\Delta V_{o}$ are the internal and external volumetric distortion, separately, and can be

(2) $$\Delta V_{i}=\pi {\left(R_{i}+\Delta R-\frac{\Delta t}{2}\right)}^{2}\left(L+\Delta L\right)-\pi R_{i}^{2}L\approx \pi R_{i}L\left(2R_{m}\varepsilon_{2}-t\varepsilon_{3}+R_{i}\varepsilon_{1}\right)$$

(3) $$\Delta V_{o}=\pi {\left(R_{o}+\Delta R+\frac{\Delta t}{2}\right)}^{2}\left(L+\Delta L\right)-\pi R_{o}^{2}L\approx \pi R_{o}L\left(2R_{m}\varepsilon_{2}+t\varepsilon_{3}+R_{o}\varepsilon_{1}\right)$$

where $R_{i}$ and $R_{o}$ are the internal and external radius of cylindrical layer separately; $R_{m}$ is the average radius; *L* is the initial length of the layer, *t*; and Δt is the thickness and thickness deformation of the cylindrical layer; $\varepsilon_{1}$, $\varepsilon_{2}$ and $\varepsilon_{3}$ are the strains of the cylindrical layer, which are defined by the following equation:

(4) $$\left[\begin{array}{c} \varepsilon_{1}\\ \varepsilon_{2}\\ \varepsilon_{3} \end{array}\right]=\left[\begin{array}{c} \frac{\Delta L}{L}\\ \frac{\Delta R}{R_{m}}\\ \frac{\Delta t}{t} \end{array}\right]$$

Thus, Equation (1) can be rewritten as follows:

(5) $$W=\left(F\varepsilon_{1}+T\gamma \right)L+\pi P_{i}R_{i}L\left(2R_{m}\varepsilon_{2}-t\varepsilon_{3}+R_{i}\varepsilon_{1}\right)-\pi P_{o}R_{o}L\left(2R_{m}\varepsilon_{2}+t\varepsilon_{3}+R_{o}\varepsilon_{1}\right)$$

where γ=Δφ/L.

According to the above strains, the derivation of the work carried out by the external axisymmetric loads yields the following [34,35]:

(6) $$\partial W=\left[\begin{array}{c} \frac{\partial W}{\partial \varepsilon_{1}}\\ \frac{\partial W}{\partial \gamma }\\ \frac{\partial W}{\partial \varepsilon_{2}}\\ \frac{\partial W}{\partial \varepsilon_{3}} \end{array}\right]=\left[\begin{array}{c} \left(F+\pi P_{i}R_{i}^{2}-\pi P_{o}R_{o}^{2}\right)L\\ TL\\ \left(2\pi P_{i}R_{i}R_{m}-2\pi P_{o}R_{o}R_{m}\right)L\\ \left(-\pi P_{i}R_{i}t-\pi P_{o}R_{o}t\right)L \end{array}\right]$$

For anisotropic material, the relationship between the strain and stress based on Hooke’s law can be given by the following equation:

(7) $$\begin{array}{l} \sigma_{1}=\frac{\mu E}{\left(1+\mu \right)\left(1-2\mu \right)}\left(\varepsilon_{1}+\varepsilon_{2}+\varepsilon_{3}\right)+2G\varepsilon_{1}\\ \sigma_{2}=\frac{\mu E}{\left(1+\mu \right)\left(1-2\mu \right)}\left(\varepsilon_{1}+\varepsilon_{2}+\varepsilon_{3}\right)+2G\varepsilon_{2}\\ \sigma_{3}=\frac{\mu E}{\left(1+\mu \right)\left(1-2\mu \right)}\left(\varepsilon_{1}+\varepsilon_{2}+\varepsilon_{3}\right)+2G\varepsilon_{3}\\ \tau =\frac{E}{2\left(1+\mu \right)}\gamma \end{array}$$

where $\sigma_{1}$, $\sigma_{2}$, $\sigma_{3}$ and τ are the corresponding stresses of each direction; *E* is the Young’s modulus and μ is the Poisson’s ratio.

The strain energy *U* can be presented using the following equation:

(8) $$\begin{array}{l} U=\frac{1}{2}\int_{V}\left(\sigma_{1}\varepsilon_{1}+\sigma_{2}\varepsilon_{2}+\sigma_{3}\varepsilon_{3}+\tau_{12}\gamma_{12}\right)dV\\ \;\;=\frac{1}{2}\int_{V}\left[\left(\lambda +2G\right)\left(\varepsilon_{1}^{2}+\varepsilon_{2}^{2}+\varepsilon_{3}^{2}\right)+2\lambda \left(\varepsilon_{1}\varepsilon_{2}+\varepsilon_{1}\varepsilon_{3}+\varepsilon_{2}\varepsilon_{3}\right)+GR_{m}^{2}\gamma^{2}\right]dV \end{array}$$

Substituting the volume of the cylindrical layer $V=\pi \left(R_{o}^{2}-R_{i}^{2}\right)L$ and applying the partial derivatives for each strain yields the following:

(9) $$\begin{array}{l} \frac{\partial U}{\partial \varepsilon_{1}}=\left[\left(\lambda +2G\right)\varepsilon_{1}+\lambda \left(\varepsilon_{2}+\varepsilon_{3}\right)\right]AL\\ \frac{\partial U}{\partial \gamma }=\pi GR_{m}^{2}\gamma \left(R_{o}^{2}-R_{i}^{2}\right)L\approx \pi G\gamma \left(R_{o}^{4}-R_{i}^{4}\right)L\\ \frac{\partial U}{\partial \varepsilon_{2}}=\left[\left(\lambda +2G\right)\varepsilon_{2}+\lambda \left(\varepsilon_{1}+\varepsilon_{3}\right)\right]AL\\ \frac{\partial U}{\partial \varepsilon_{3}}=\left[\left(\lambda +2G\right)\varepsilon_{3}+\lambda \left(\varepsilon_{1}+\varepsilon_{2}\right)\right]AL \end{array}$$

where

$$\begin{array}{l} \lambda =\frac{\mu E}{\left(1+\mu \right)\left(1-2\mu \right)}\\ G=\frac{E}{2\left(1+\mu \right)}\\ A=\pi \left(R_{o}^{2}-R_{i}^{2}\right) \end{array}$$

Based on the functional principle, the relationship between the total potential energy and the work carried out by the external loads and internal energy can be given by the following:

(10) ∂Π=∂W−∂U=0

The equilibrium equation for the cylindrical layer can be obtained using Equations (6), (9) and (10) (Ren et al., 2014 [23]):

(11) $$\left[\begin{array}{c} k_{11}\;\;k_{12}\;\;k_{13}\;\;k_{14}\\ k_{21}\;\;k_{22}\;\;k_{23}\;\;k_{24}\\ k_{31}\;\;k_{32}\;\;k_{33}\;\;k_{34}\\ k_{41}\;\;k_{42}\;\;k_{43}\;\;k_{44} \end{array}\right]\left[\begin{array}{c} \Delta L/L\\ \Delta \phi /L\\ \Delta R/R_{m}\\ \Delta t/t \end{array}\right]=\left[\begin{array}{c} F+\pi P_{i}R_{i}^{2}-\pi P_{o}R_{o}^{2}\\ T\\ 2\pi R_{m}\left(P_{i}R_{i}-P_{o}R_{o}\right)\\ -\pi t\left(P_{i}R_{i}+P_{o}R_{o}\right) \end{array}\right]$$

where

$$k_{11}=\frac{\mu EA}{\left(1+\mu )(1-2\mu \right)}+\frac{EA}{1+\mu }\;k_{13}=k_{31}=\frac{\mu EA}{\left(1+\mu )(1-2\mu \right)}\;k_{22}=\frac{E}{2(1+\mu )}.\frac{\pi }{2}(R_{o}^{4}-R_{i}^{4})\;k_{24}=k_{42}=0\;k_{34}=k_{43}=\frac{\mu EA}{\left(1+\mu )(1-2\mu \right)}\;k_{12}=k_{21}=0\;k_{14}=k_{41}=\frac{\mu EA}{\left(1+\mu )(1-2\mu \right)}\;k_{23}=k_{32}=0\;k_{33}=\frac{\mu EA}{\left(1+\mu )(1-2\mu \right)}+\frac{EA}{1+\mu }\;k_{44}=\frac{\mu EA}{\left(1+\mu )(1-2\mu \right)}+\frac{EA}{1+\mu }$$

### 2.2. Theoretical Model of Helical Layer under Axisymmetric Loads

**Figure 3:**
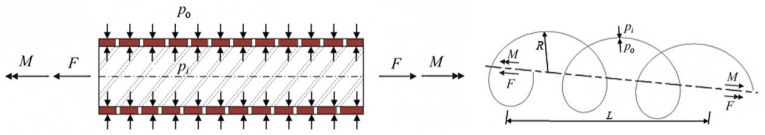
Force schematic diagram of the helical layer.

For the helical layer, assuming that the tendon can only deform along its axial direction, the sketch of a loaded helical tendon is presented in Figure 3. Based on the geometric properties of the helical tendon, the axial strain $\varepsilon_{a}$ and the radial strain $\varepsilon_{r}$ can be defined as follows [7,9,35]:

(12) $$\begin{array}{l} \varepsilon_{a}=\cos^{2}\alpha \frac{\Delta L}{L}+R_{m}\sin\alpha \cos\alpha \frac{\Delta \varphi }{L}+\sin^{2}\alpha \frac{\Delta R}{R_{m}}\\ \varepsilon_{r}=\frac{\Delta t}{t} \end{array}$$

The strain energy of a single helical tendon is calculated as follows [6]:

(13) $$U_{s}=\frac{n}{2}\int_{V}\left(\sigma_{a}\varepsilon_{a}+\sigma_{r}\varepsilon_{r}\right)dV$$

where *n* is the number of helical tendons and $\sigma_{a}$ and $\sigma_{r}$ are the corresponding stresses of the helical tendon.

Similar to the deduction process in Section 2.1, the final equilibrium equation for the helical layer can be presented as follows [6,9,28]:

(14) $$\left[\begin{array}{cccc} k_{11} & k_{12} & k_{13} & k_{14}\\ k_{21} & k_{22} & k_{23} & k_{24}\\ k_{31} & k_{32} & k_{33} & k_{34}\\ k_{41} & k_{42} & k_{43} & k_{44} \end{array}\right]\left[\begin{array}{c} \Delta L/L\\ \Delta \phi /L\\ \Delta R/R_{m}\\ \Delta t/t \end{array}\right]=\left[\begin{array}{c} F+\pi P_{i}R_{i}^{2}-\pi P_{0}R_{0}^{2}\\ T\\ 2\pi R_{m}(P_{i}R_{i}-P_{0}R_{0})\\ -\pi t(P_{i}R_{i}+P_{0}R_{0}) \end{array}\right]$$

where

$$\begin{array}{l} k_{11}=\frac{nEA}{1-\mu^{2}}\cos^{3}\alpha \\ k_{13}=k_{31}=\frac{nEA}{1-\mu^{2}}\sin^{2}\alpha \cos\alpha \\ k_{22}=\frac{nEAR_{m}^{2}}{1-\mu^{2}}\sin^{2}\alpha \cos\alpha \\ k_{24}=k_{42}=\frac{nEAR_{m}\mu }{1-\mu^{2}}\sin\alpha \\ k_{34}=k_{43}=\frac{nEA\mu }{1-\mu^{2}}\frac{\sin^{2}\alpha }{\cos\alpha } \end{array}\begin{array}{l} k_{12}=\frac{nEAR_{m}}{1-\mu^{2}}\sin\alpha \cos^{2}\alpha \\ k_{14}=k_{41}=\frac{nEA\nu }{1-\mu^{2}}\cos\alpha \\ k_{23}=k_{32}=\frac{nEAR_{m}}{1-\mu^{2}}\sin^{3}\alpha \\ k_{33}=\frac{nEA}{1-\mu^{2}}\frac{\sin^{4}\alpha }{\cos\alpha }\\ k_{44}=\frac{nEA}{(1-\mu^{2})\cos\alpha } \end{array}$$

### 2.3. Geometric Relationship between Adjacent Layers

By combining the equilibrium equations of cylindrical layers and helical layers within an unbonded flexible riser model, the relationship between the external force and strains can be established, although it is still impossible to solve all the unknowns. Thus, the geometric relationship and contact between the adjacent layers are also considered. Based on the restriction of geometric continuity, the interlayer contact relationship between adjacent layers is given by the following equation:

(15) $$R_{m}^{j}+\Delta R^{j}+\frac{t^{j}+\Delta t^{j}}{2}=R_{m}^{j+1}+\Delta R^{j+1}-\frac{t^{j+1}+\Delta t^{j+1}}{2}\;$$

where *j* stands for the layer’s number.

## 3. Numerical Simulation

**Figure 4:**
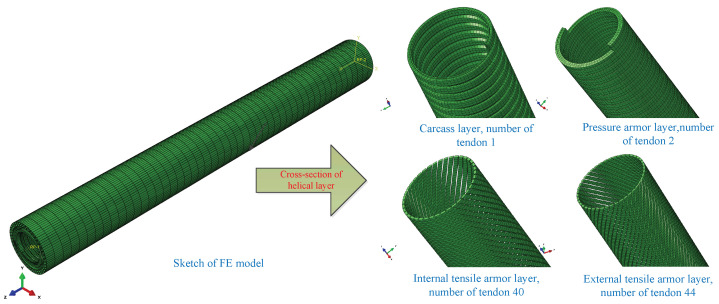
Sketch of finite element model of unbonded flexible riser.

Data for unbonded flexible riser models are rare, since the production of unbonded flexible risers is in the hands of only a few riser manufacturers. The detailed geometric and material properties of unbonded flexible riser models are very limited, except for Witz’s experiment [36]. Witz presented some classic experiments based on an eight-layer 2.5-inch unbonded flexible riser model, which not only included detailed properties of an unbonded flexible riser, but also detailed experimental measurements and boundary conditions, and many scholars have conducted research on the mechanical properties of unbonded flexible risers based on these tests since then. The corresponding geometric and material characteristics are presented in Table 1. A sketch of the corresponding numerical model, including the all-inclusive helical layer, is illustrated in Figure 4.

Before the numerical simulation, some settings are first drawn. First of all, the numerical model should have a sufficient length to reduce the effect of stress concentration on end boundary conditions, typically more than twice the tensile armor layer’s pitches [21]. According to Table 1, the length of an unbonded flexible riser model is 1.0 m, which is about twice the tensile armor layer’s pitches. To simulate the deformation of each layer in detail, solid elements are applied and all interlayers are meshed into eight-node linear brick elements with incompatible modes, and the grid independence has been verified. Since the geometric nonlinearity is strong within the riser model, the explicit solution method is applied to avoid non-convergence in the numerical simulation. Since it is difficult to define the accurate contact between and within interlayers, general contact is thus applied to define the nonlinear contact, where the tangential behavior is simulated by using the Coulomb friction model [25] and the corresponding frictional coefficient is 0.1 [37], while the normal contact is set to hard contact. Two reference points, RP1 and RP2 (see Figure 4), are set at the geometric center of the top and end cross-section and all the layer’s nodes at the edge of the cross-section are kinematically and rigidly bounded onto the two reference points, thus applying the boundary conditions and external loads. Among them, all degrees of freedom on the bottom RP2 are constrained, and in addition, the axial tensile degree of the top RP1 along the Z-axis is free. For axial tensile analysis, a 500 kN axial tension is applied on the RP1 reference point. Meanwhile, for the ultimate stress analysis, a sufficiently large axial displacement is applied and the corresponding axial tension is from the RP2 reference point. The inertia effect must be taken into account for the explicit solution method and the kinetic energy during the calculation process should be controlled. As a consequence, the ratio of kinetic energy (ALLKE) to internal energy (ALLIE) must be kept under 5%; thus, the smooth step loading method is applied to decrease the inertia effect. Thus, a quasi-static loading method increasing the loading time is applied to eliminate the influence of the inertia effect. To accelerate the simulation time, the mass scaling method is applied in this paper, and the minimum loading time step of $2\times 10^{-7}$ is small enough to ensure calculation accuracy. The common Ramberg–Osgood model is applied to describe the nonlinear stress of tensile armor layers, considering the elasticity and plasticity of the material.

## 4. Model Verification

**Figure 5:**
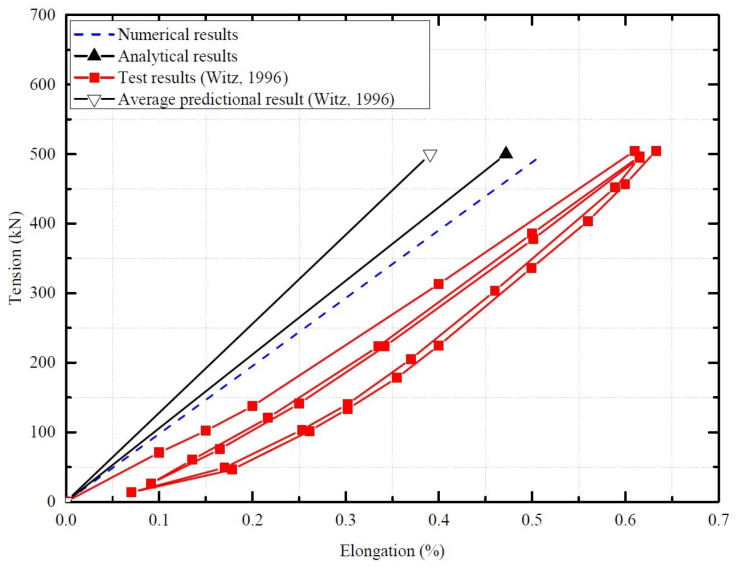
Axial tension–elongation curves of different methods [36].

**Figure 6:**
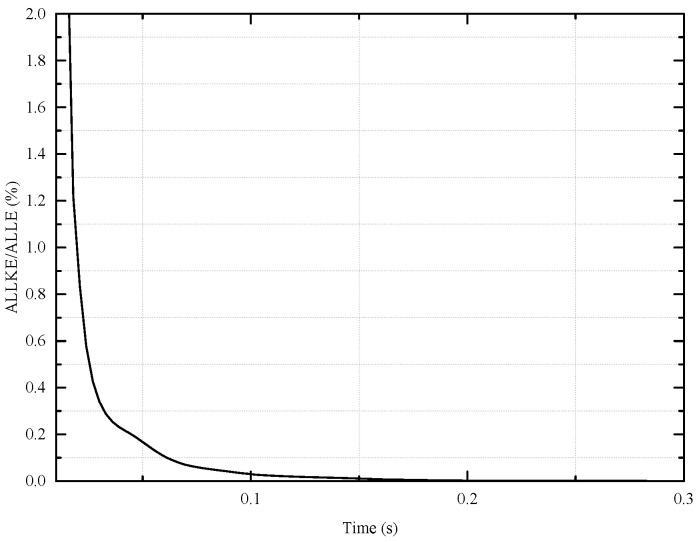
Ratio of kinetic energy to internal energy.

Based on Witz’s test [36], the proposed numerical and theoretical methods are verified according to the tensile behavior of the unbonded flexible riser. The boundary condition is set as Witz’s experiment. One end (reference point RP2) is set as fixed; the other end is set as top end free (reference point RP1). And the external axial tension is also set at reference point RP1, of which the total axial tension is 500 kN. The quasi-static method is applied to minimize the inertia effect during the numerical simulation. The axial tension–elongation curves of different methods are illustrated in Figure 5, and the corresponding tensile stiffness is presented in Table 2. During the numerical simulation, the ratio of kinetic energy to internal energy was as presented in Figure 6, where the ratio is no more than 5%.

In Figure 5, the black line, the blue line and the red line stand for theoretical, numerical and test results, respectively, and the average prediction results by the manufactures and academics are also presented. The numerical and theoretical results do not exhibit significant nonlinearities, since the tensile armor layers do not have obvious slippage. As for the axial tensile stiffness, and as can be seen in Table 1, compared with the experimental results, the relative deviations of the theoretical results and numerical results are 14.18% and 8.07%, respectively. The relative deviation is mainly caused by the following reasons: firstly, the nonlinear contact and the radial and circumferential deformation within helical layers with complex cross-sections, like carcass and pressure armor layers, cannot be included at the moment; secondly, the separate interlayers are assumed to present distributed stress along the length of the riser, which cannot occur due to the end effect; and, finally, the hypothesis of different interlayers with the same axial displacement and twist per unit of length can only be validated at the two end sections in the experimental and FE models. All the presenting assumptions made in the analytical method make up the relative deviation, but the analytical prediction in this paper is still better than the average prediction by other institutions and scholars (see Figure 5 [34]). Indeed, the numerical results are relatively close to the experimental results. The proposed numerical method in this paper includes all the geometric and contact nonlinearity within the unbonded flexible riser model, and can properly predict the axial tensile stiffness, where the relative deviations are mainly caused by the possible initial pre-stress and initial defects in the unbonded flexible riser specimens [36].

## 5. Discussion

Based on the proposed theoretical and numerical methods, in consideration of the elastoplasticity of the tensile armor layer, the fracture failure is studied in this section. Additionally, considering the effect of the frictional coefficient, the corresponding axial stiffness is analyzed.

### 5.1. Fracture Failure of Tensile Armor Layer under Axial Tension

The carbon steel material is modeled by using an ideal elastic–plastic model, with the yield stress at 650 MPa and the tangent modulus at 1172.58 MPa [1].

For the theoretical method, the fracturing of the tensile armor layer occurs when it reaches yield stress. And the corresponding stress can be calculated as follows:

(16) $$\begin{array}{l} \sigma_{a}=\frac{E}{1-\mu^{2}}\left(\varepsilon_{a}+\mu \varepsilon_{r}\right)\\ \sigma_{r}=\frac{E}{1-\mu^{2}}\left(\varepsilon_{r}+\mu \varepsilon_{a}\right) \end{array}$$

For the numerical method, except for the tensile armor layer, all the other layers have set material with liner elasticity. Due to the special structural form of the carcass layer and the pressure armor layer, the fracture failure of these two layers generally occurs at large axial elongation (about 3.5%), which is much higher than the axial elongation at which the tensile armor layer of the unbonded flexible riser fails. Therefore, the failure of the self-locking structure of these two layers is not considered in this section.

**Figure 7:**
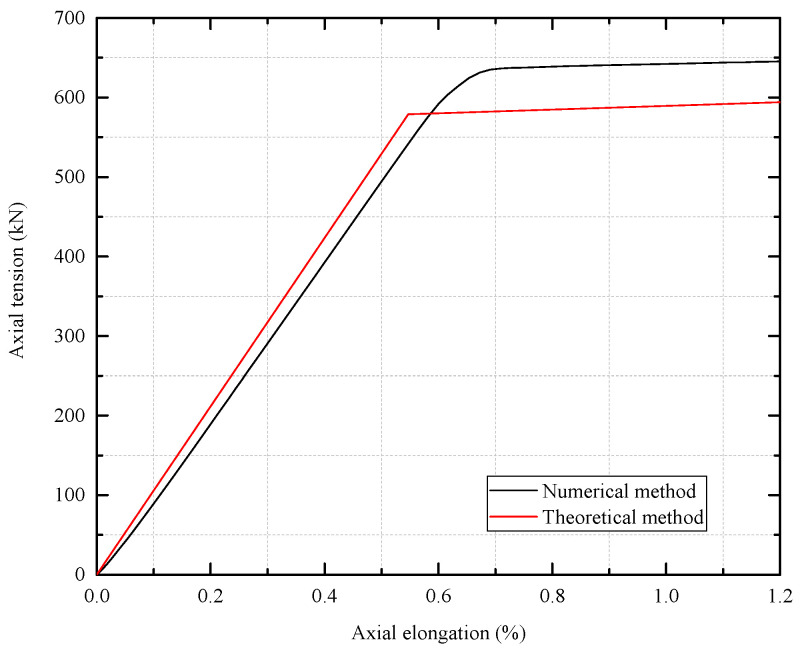
Axial tension–elongation curves with nonlinearity of materials.

Figure 7 presents the axial tension–axial elongation curves of the unbonded flexible riser model when considering the elasticity and plasticity of the materials. It can be seen that the axial bearing capacity obtained by the two methods is comparable. 

**Figure 8:**
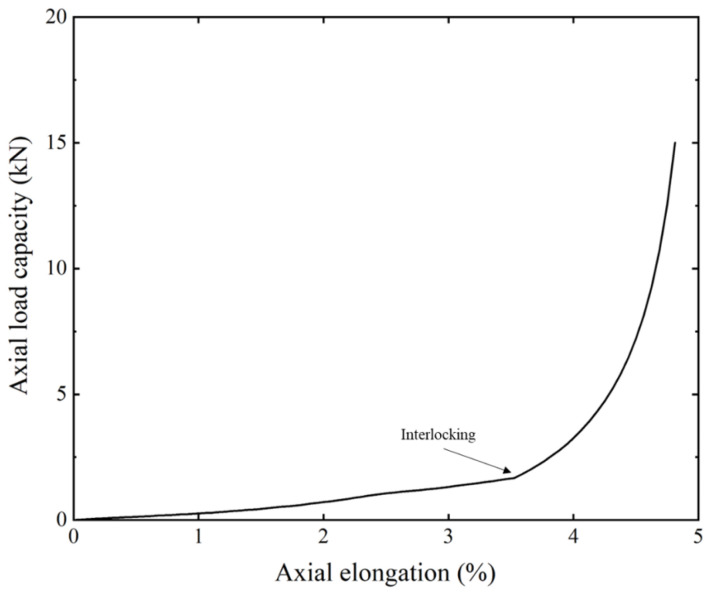
Axial tension–elongation curves of carcass layer.

For comparison reasons, the effect of the self-locking layers is also considered in this paper. The carcass layer and pressure armor layer in an unbonded flexible riser are typically set to resist the internal and external pressure and prevent the riser’s collapse, with a low ability to carry axial tension. As the two layers are geometrically similar, only the axial tensile elongation of a single carcass layer is presented in Figure 8. As can be seen in the linear stage of Figure 8, the axial elongation of the carcass layer increases linearly with the increasing axial tension; however, this is with a relatively low axial tensile stiffness, which is no more than 1% of the overall axial tensile stiffness of an unbonded flexible riser (Table 2). When the axial tensile elongation is about 3.5%, the carcass layer becomes interlocking and the axial tensile stiffness sees some strengthening, but the corresponding axial tensile elongation far exceeds the axial elongation when fracture failure occurs in the tensile armor layer, without affecting the numerical simulation results.

Theoretically, when the axial force is 578.84 kN, the internal tensile armor layer model reaches the yield stress, and then the axial tensile stiffness decreases sharply to 2.34 MN, which is only 2.21% of the original axial tensile stiffness. And the external tensile armor layer also reaches the yield stress when the axial tension is 613.71 kN, and then the axial tensile stiffness of the unbonded flexible riser decreases further to 1.53 MN. The numerical method shows some nonlinearity due to the fact that the stresses in the tensile armor layer structure along the pipe length direction are not uniformly distributed.

**Figure 9:**
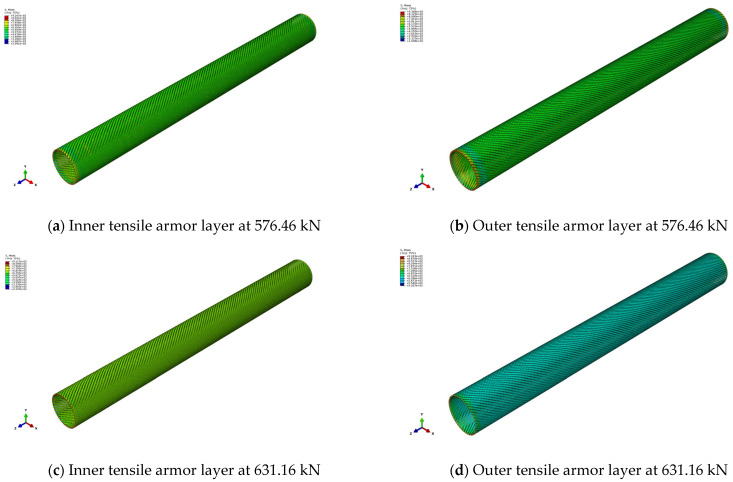
Stress contours of unbonded flexible riser under different axial tensions.

Figure 9 gives the Von Mises stress of the internal and external tensile armor layers under different axial tensions, and the internal tensile armor layer shows a higher stress than the external tensile armor layer. Figure 9a,b give the stress contours of the internal and external tensile armor layers at an axial tension 576.46 kN. At this time, the stress distribution in the middle part of the internal tensile armor layer is about uniformly distributed when neglecting the end boundary effect, and there are some elements reaching yield stress undergoing plastic deformation and presenting a strip-like distribution. And the external tensile armor layer structure and the rest of the units, in addition to the part of the element near the boundary in the plastic stage, are still in the elastic range. Figure 9c,d give the stress contours of the internal and external tensile armor layers under an axial tension of 631.16 kN, and it can be seen that, at this time, the unit of the internal and external tensile armor layers completely enters into the plastic stage, and it can be regarded that the unbonded flexible riser arrives at its ultimate load carrying capacity. And the numerical perdition result matches well with the theoretical prediction, and the relative deviation is only 2.76%.

### 5.2. Effect of Frictional Coefficient on the Axial Tensile Stiffness

An unbonded flexible riser would produce axial deformation under the action of axial tension, in which the tendon’s deformation is along its own axial change and would have relative slip with the adjacent cylindrical shell layer, producing a certain amount of friction energy. Meanwhile, due to the carcass layer and pressure armor layer of such a self-locking structure itself in the occurrence of axial deformation, this would also occur in the complex inter-contact effect and produce a certain amount of friction energy. When the friction coefficient changes, it would have a certain effect on the corresponding friction energy. Since the theoretical model fails to take into account the friction energy generated by the relative slip between and within layers, the influence of different friction coefficients is analyzed by numerical methods.

**Figure 10:**
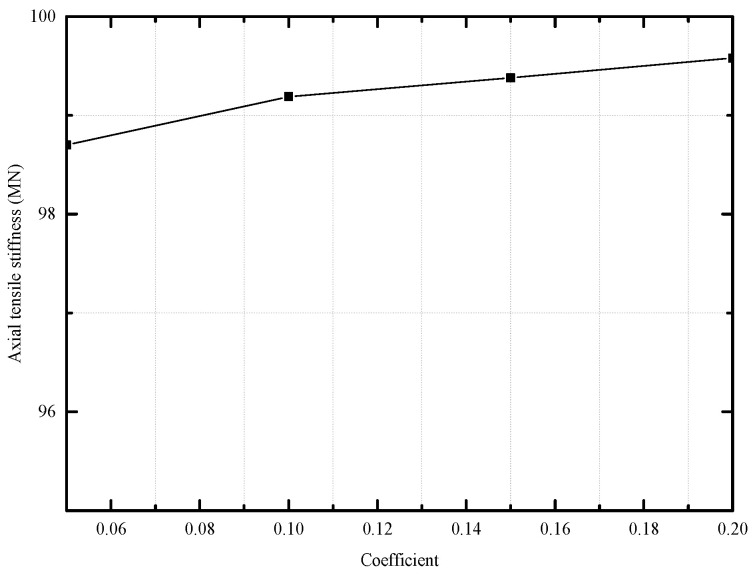
Axial tensile stiffness with different friction coefficients.

Considering that the friction coefficient varies from 0.05 to 0.20, the axial tensile stiffness of unbonded flexible risers with different friction coefficients is given by numerical methods at friction coefficient intervals of 0.05. Since all the loading cases present nearly positive proportional growth when neglecting the instability of numerical simulations, only the corresponding axial tensile stiffness is shown in Figure 10, which reveals that the axial tensile stiffness of the unbonded flexible riser model increases with the increasing of the friction coefficient. However, the axial stiffness of unbonded flexible risers with friction coefficients is not sensitive to the friction coefficient, and the axial tensile stiffness increases by less than 1% when the friction coefficient increases by three times. Meanwhile, it can be seen that the larger the interlayer friction coefficient, the closer the numerical results are to the theoretical results. This is because the theoretical model is based on the infinite friction coefficient (no relative slip between layers), so the larger the numerical results, the closer they are to this assumption, and the relative deviation becomes smaller.

At the same time, by the numerical method for completely ignoring the friction between and within layers (i.e., the friction coefficient is equal to 0), the axial tensile stiffness decreases sharply, only 83.29 MN, which deviates from the theoretical and experimental results; the tensile armored layer loses the mutual constraints between the layers, and it can slip freely between the layers, and the axial tensile stiffness decreases, so the effect of completely ignoring friction would lead to the calculation of the numerical results not being accurate.

## 6. Conclusions

This paper presents the axial tensile behavior of unbonded flexible risers and the fracture failure of the tensile armor layer under external axial force. Firstly, the theoretical model was derived by the function principle. Then, based on a typical 2.5-inch eight-layer unbonded flexible riser model, the corresponding numerical model, in full consideration of the geometric properties and complex contact, was established within the ABAQUS software. After verification through Witz’s experimental case, the proposed numerical and theoretical methods can possibly predict the behavior of unbonded flexible risers. Finally, the behavior of unbonded flexible risers under axial loads is conducted using the proposed methods. Some conclusions are drawn at the end of this paper:

1. The self-locking structures of the carcass layer and pressure armor layer have little effect on the axial tensile stiffness and axial tensile strength of an unbonded flexible riser. The proposed numerical method, due to its ability to model the complex geometric and contact behavior within unbonded flexible risers, is closer to the test results compared to the analytical results. But there is still a relative deviation of 8.07% between the numerical and the experimental results, since there always exists initial pre-stress in the actual riser due to the manufacturing process, and the detailed frictional coefficient is also unknown for the experimental measurement.
2. In the inner tensile armor layer, the fracture would occur first before the outer tensile armor layer. The numerical method agrees well with the theoretical method, and the corresponding relative deviation is only 2.76%. The axial tensile stiffness of an unbonded flexible riser model would decrease sharply when fracture occurs in the tensile armor layer, and the unbonded flexible riser loses bearing capacity when both tensile armor layers reach yield stress.
3. The effect of the friction coefficient on the tensile behavior can well explain the relative deviations of numerical, analytical and experimental methods. The increasing friction coefficient can improve the axial tensile stiffness of an unbonded flexible riser. When the friction coefficient is infinite, the numerical simulation remains consistent with the theoretical assumption that no interlayer slip occurs, and the larger the friction coefficient, the closer it is to the theoretical result. When completely neglecting the interlayer friction, the helical tendon can move freely under the external axial tension, similar to the hysteresis during test measurements, and the corresponding axial tensile stiffness is also consistent with the test results.

## Figures and Tables

**Table 1:** Geometric and material properties of unbonded flexible riser model.

Layer Number	Layer Type	Section Size (mm^2^)	Number of Tendons	Inner Radius (mm)	Outer Radius (mm)	Laying Angle (°)	Material	Young’s Modulus (GPa)	Poisson’s Ratio
1	Carcass	19.60	1	31.60	35.10	−87.5	AISI 304	205	0.29
2	Pressure sheath	-	-	35.10	40.00	-	Nylon 12	0.28	0.30
3	Zeta layer	5.55	1	40.05	46.25	−85.5	FI-15	205	0.29
4	Anti-friction layer	-	-	46.25	47.75	-	Nylon 11	0.30	0.30
5	Inner tensile armor layer	18.00	40	47.75	50.75	−35.0	FI-41	205	0.29
6	Anti-friction layer	-	-	50.75	52.25	-	Nylon 11	0.30	0.30
7	Outer tensile armor layer	18.00	44	52.25	55.25	35.0	FI-41	205	0.29
8	Fabric tape	4.50	-	55.25	55.75	-	-	0.60	0.30

**Table 2:** Axial stiffness by different methods.

Method	Axial Tensile Stiffness (MN)	Relative Deviation (%)
Analytical method	105.88	14.18
Numerical method	99.19	8.07
Experimental method	91.19	-

## Data Availability

Data are contained within the article.
